# Time evolution of the hierarchical networks between PubMed MeSH terms

**DOI:** 10.1371/journal.pone.0220648

**Published:** 2019-08-12

**Authors:** Sámuel G. Balogh, Dániel Zagyva, Péter Pollner, Gergely Palla

**Affiliations:** 1 Dept. of Biological Physics, Eötvös University, Budapest, Hungary; 2 MTA-ELTE Statistical and Biological Physics Research Group, Budapest, Hungary; Universidad Rey Juan Carlos, SPAIN

## Abstract

Hierarchical organisation is a prevalent feature of many complex networks appearing in nature and society. A relating interesting, yet less studied question is how does a hierarchical network evolve over time? Here we take a data driven approach and examine the time evolution of the network between the Medical Subject Headings (MeSH) provided by the National Center for Biotechnology Information (NCBI, part of the U. S. National Library of Medicine). The network between the MeSH terms is organised into 16 different, yearly updated hierarchies such as “Anatomy”, “Diseases”, “Chemicals and Drugs”, etc. The natural representation of these hierarchies is given by directed acyclic graphs, composed of links pointing from nodes higher in the hierarchy towards nodes in lower levels. Due to the yearly updates, the structure of these networks is subject to constant evolution: new MeSH terms can appear, terms becoming obsolete can be deleted or be merged with other terms, and also already existing parts of the network may be rewired. We examine various statistical properties of the time evolution, with a special focus on the attachment and detachment mechanisms of the links, and find a few general features that are characteristic for all MeSH hierarchies. According to the results, the hierarchies investigated display an interesting interplay between non-uniform preference with respect to multiple different topological and hierarchical properties.

## Introduction

In the recent decades the network approach has become fundamental in the studies of various phenomena in nature and society, ranging from the level of interactions within cells to the level of the Internet, economic networks, and the society [1, 2]. A very important topic in this field is related to the hierarchical organization of networks [3–7]. Grasping the signs of hierarchy in networks is a non-trivial task with a number of possible different approaches, including the statistical inference of an underlying hierarchy based on the observed network structure [4], and the introduction of various hierarchy measures [8–13]. Examples of empirical studies on hierarchical networks are including the transcriptional regulatory network of Escherichia coli [14], the dominant-subordinate hierarchy among crayfish [15], the leader-follower network of pigeon flocks [16, 17] and harems of Przewalski horses [18], the rhesus macaque kingdoms [19], neural networks [20] and technological networks [5], scientific journals [21], social interactions [22–25], urban planning [26, 27], on-line news content [28], ecological systems [29, 30], and evolution [31–33]. In addition, hierarchical organisation is also related to the non-normality of networks [34], the topological properties of various scientific and techno-scientific fields [21, 35–39] (usually depicted by citation networks), and the optimal performance of interacting agent groups [40–42]. Hierarchies are usually depicted as directed acyclic graphs, in which the links are not allowed to form directed cycles, and where a pair of nodes connected by a link are assumed to be in some sort of asymmetric relationship with each other such as parents and children, leaders and followers, etc.

Networks representing real systems are subject to constant evolution in most of the cases, and some relevant aspects of the laws forming the shape of networks changing over time have already been uncovered in the scientific literature. Probably most famous is the preferential attachment rule for growing scale-free networks, which is one of the key concepts of the Barabási-Albert model [43], and was detected also by empirical studies of network data [44–47]. Another notable example is provided by the studies of the various statistical features of community evolution in networks [48]. Along the same line, in the present paper our aim is to examine the statistical properties of time dependent networks with a hierarchical structure.

Our study is based on the data provided by the NCBI about the MeSH terms, which were introduced for helping the search in the PubMed publication database of the NCBI (comprising more than 29 million citations for biomedical literature) at various levels of specificity. The MeSH terms are hierarchically organized: At the most general level of the hierarchical structure we find very broad headings such as “Organisms” or “Information Science”, whereas more specific headings are found at deeper (more narrow) levels. Due to the rapidly developing nature of the medical-, biochemical- and biological sciences, the set of available MeSH terms are yearly updated by the curators of PubMed. This provides a fascinating empirical data-set for the study of time dependent hierarchical networks. A few previous studies on this data-set have already been published, approaching the development of the MeSH term hierarchies from an ontological perspective [49–52]. The main focus of these results was on the growth of the system, concentrating on how are the newly introduced MeSH terms categorized and linked under already existing older MeSH terms. In our present study we show that restructuring plays an equally important role in forming the structure of the MeSH hierarchies.

Our goal is to examine the statistical features of the time evolution in the observed hierarchies. One of the central questions we are interested in is how do the different topological- and hierarchical properties of the nodes influence the attachment and detachment of links during the restructuring. Understanding the nature of these processes can help the creation of hierarchy evolution models that can predict which part of the hierarchy is most likely to be rewired in the future, and what is the expected change in the overall features of the hierarchy.

## Data and methods

### Basic properties of the MeSH hierarchies

The directed networks we consider are based on the classifications provided by PubMed, specifying at least one parent for any available MeSH term, except for the roots of the hierarchies. The raw data we use is publicly available on the link provided in Ref. [53]. There are altogether 16 different roots, and the total number of descendants of the individual roots (the sizes of the hierarchies) varies roughly between a 1,00 and a 10,000 nodes, whereas the time span of our analysis is 14 years. In Table 1 we list a few basic properties of these networks, including the minimum and maximum sizes, the maximum level depth and the average fraction of changed links under one time step (one year). In S1 Text we also provide more detailed tables listing the yearly size of the hierarchies, together with the number of added and deleted nodes and links.

**Table 1:** Basic hierarchy data.

	Root name	size range	max. depth	average change
A	Anatomy	1350–1826	10	4.66%
B	Organisms	2252–3815	13	6.49%
C	Diseases	3975–4799	8	4.23%
D	Chemicals and Drugs	6902–9934	11	6.22%
E	Analytical, Diagnostic and Therapeutic Techniques and Equipment	2040–2924	9	4.89%
F	Psychiatry and Psychology	807–1083	7	3.60%
G	Phenomena and Processes	1733–2259	10	15.18%
H	Disciplines and Occupations	334–537	8	12.07%
I	Anthropology, Education, Sociology and Social Phenomena	449–641	9	5.23%
J	Technology, Industry, Agriculture	254–582	10	8.92%
K	Humanities	152–200	7	3.93%
L	Information Science	322–476	9	5.82%
M	Named Groups	174–290	7	5.71%
N	Health Care	1072–1795	10	4.94%
V	Publication Characteristics	137–163	6	3.44%
Z	Geographicals	369–402	6	1.94%

The 1^st^ column lists the hierarchy ID, the 2^nd^ gives the name of the root, the 3^rd^ column provides the minimum and maximum sizes during the time evolution, the 4^th^ contains the maximum level depth, and finally the 5^th^ column lists the average fraction of changed links under one year.

**Fig 1:**
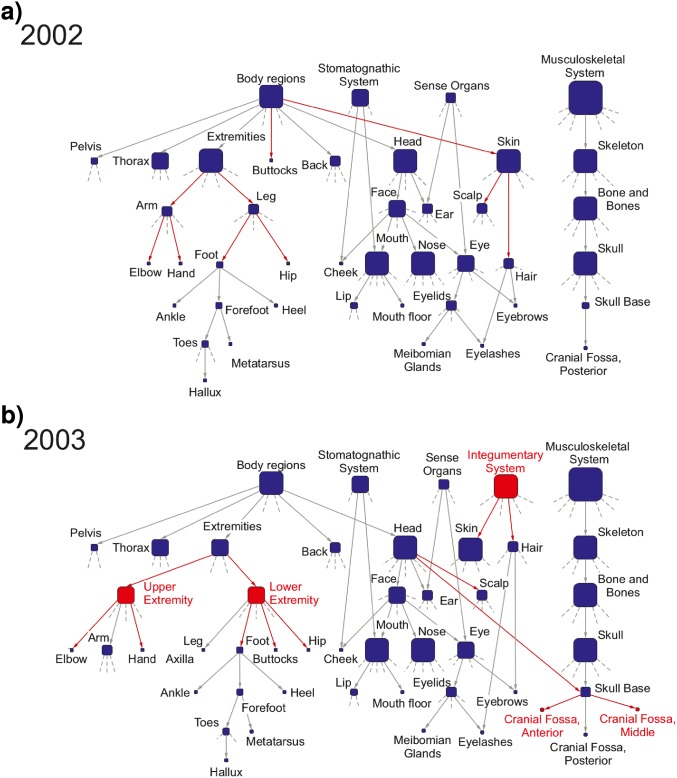
Changes between subsequent time steps in a MeSH hierarchy. a) A small part of the hierarchy ‘A’ (Anatomy) in 2002. Red links are deleted in the next time step b) The corresponding part of the same hierarchy in 2003. Nodes and links colored red are newly appearing elements.

The links in our network representation are pointing from the parents to their children. Since a part of the MeSH terms have multiple parents, the studied networks are not strictly tree-like, instead they correspond to a directed acyclic graphs. Due to the yearly updates, the structure of these networks is subject to constant evolution: New MeSH terms can appear, terms becoming obsolete can be deleted or be merged with other terms, and also already existing parts of the network may be rewired. To illustrate these processes, in Fig 1. we show two snapshots from subsequent years, depicting the changes in a small subgraph from the hierarchy A (Anatomy). According to the picture, a relatively large variety of modifications can occur already in a single time step. E.g., ‘Cranial Fossa Anterior’ is a newly appearing MeSH term, which is classified under ‘Skull Base’ in Fig 1b. This type of process can be viewed in general as the growth of the hierarchy. Another intuitive process is rewiring, when both the source and the target of a newly appearing link are actually already existing (’old’) nodes, such as e.g., the new link between ‘Head’ and ‘Scalp’ in Fig 1b. Naturally, links becoming obsolete can also become deleted, as e.g., the link from ‘Body regions’ to ‘Skin’ in Fig 1a. There are also somewhat less intuitive change types as well, such as the insertion of a new node into the middle part of a branch, as e.g., the link from ‘Upper Extremity’ to ‘Arm’ in Fig 1b, or the appearance of a new link between two new nodes. A detailed classification of the possible change types is given in the Results section.

### Measuring preference during attachment or detachment

Our main focus in this paper is on the examination of possible preference with respect to various node properties during the attachment and detachment of the links. The method we use for detecting whether the attachments/detachments are uniform with respect to a given property *x*, or instead show preference towards high (or low) values of *x* is based on comparing the distribution of *x* for the chosen nodes during the change event and the distribution of *x* amongst the available nodes [23].

#### Attachment events

We begin by discussing attachment events, where (previously non existing) new links appear in the system. For simplicity let us consider first only two consecutive time steps in the data set for a single hierarchy, where we would like to examine whether the choice of nodes in the initial state is preferential or not with regard to *x*. We denote the probability distribution of *x* at the initial state by *p*(*x*), and the complementary cumulative distribution by $Q\left(x\right)=\sum_{x^{\prime }\geq x}p\left(x^{\prime }\right)$, corresponding to the fraction of nodes in the hierarchy having a property value at least as large as *x* in the initial state. In case the attachment is independent of *x*, the number of nodes chosen having a property value *x* or larger is expected to be simply proportional to *Q*(*x*). However, if larger values of *x* are preferred, then nodes having large *x* value are chosen at a higher frequency compared to what we would expect based on *Q*(*x*), and similarly, if lower values of *x* are preferred, then nodes with large *x* values are chosen at a lower frequency compared to the expectation based on *Q*(*x*). Therefore, by denoting the number of actually chosen nodes having a property value at least as large as *x* in the attachments as *w*(*x*), and taking its ratio compared to *Q*(*x*) as

$$\begin{array}{c} W\left(x\right)=\frac{w(x)}{Q(x)}, \end{array}$$ (1)

we obtain a function that is constant if the attachment is uniform in *x*, since in this case *w*(*x*) and *Q*(*x*) are simply proportional to each other for any *x*. However, if larger values of *x* are preferred, the shape of *W*(*x*) becomes increasing as a function of *x*, whereas in the opposite case, when the attachment/detachment prefers lower values of *x*, the shape of *W*(*x*) becomes decreasing.

A noteworthy property of *w*(*x*) is that for any fixed value of *x*, it follows a binomial distribution,

$$\begin{array}{c} P\left(w\left(x\right)=k\right)=\left(\begin{array}{c} A\\ k \end{array}\right)u{\left(x\right)}^{k}{\left(1-u\left(x\right)\right)}^{A-k}, \end{array}$$ (2)

where *A* is the number of attachment events, and *u*(*x*) denotes the probability for choosing a node having a property value at least as large as *x*. Simplest case is when choosing is independent of the given property, and therefore, *u*(*x*) = *Q*(*x*). If instead we assume a linear preference with regard to the studied property, *u*(*x*) can be expressed as

$$\begin{array}{c} u\left(x\right)=\sum_{i:\;x_{i}\geq x}x_{i}p\left(x_{i}\right)/\sum_{j=1}^{A}x_{j}p\left(x_{j}\right), \end{array}$$ (3)

where the summations run over the nodes in the hierarchy.

In any case, based on (2) the expected value and standard deviation of *w*(*x*) can be given as 〈*w*(*x*)〉 = *A* ⋅ *u*(*x*) and $\sigma \left(w\left(x\right)\right)=\sqrt{A\cdot u(x)(1-u(x))}$, respectively. By moving from *w*(*x*) to *W*(*x*) we obtain that according to (1) the mean and standard deviation for *W*(*x*) can be written as

$$\begin{array}{c} \left\langle W(x)\right\rangle =\frac{A\cdot u(x)}{Q(x)}, \end{array}$$ (4)

$$\begin{array}{c} \sigma (W(x))=\frac{\sqrt{A\cdot u(x)(1-u(x))}}{Q(x)}, \end{array}$$ (5)

which for an attachment process independent of *x* take the simple form of

$$\begin{array}{c} \left\langle W(x)\right\rangle =A, \end{array}$$ (6)

$$\begin{array}{c} \sigma (W(x))=\frac{\sqrt{A(1-Q(x))}}{\sqrt{Q(x)}}. \end{array}$$ (7)

**Fig 2:**
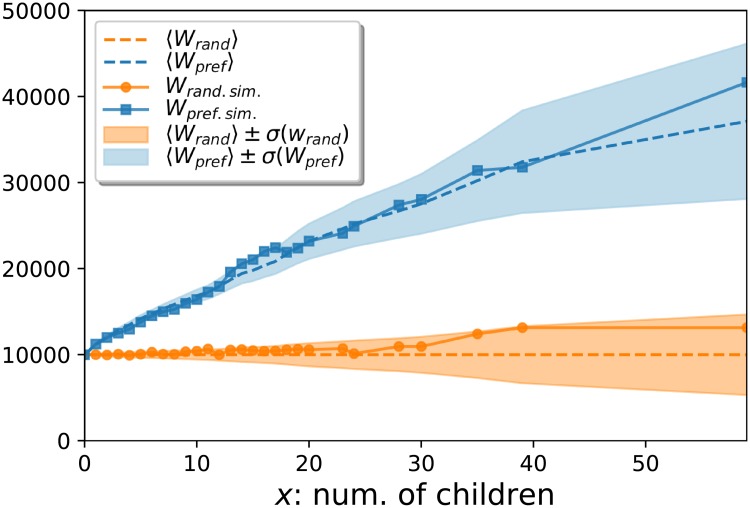
Testing *W*(*x*) by simulated attachments. The property *x* here corresponds to the number of children, and the full symbols connected by continuous lines show the measured *W*(*x*) for random attachment (independent of *x*) in orange (circles), and for preferential attachment with an additive constant (i.e. when a newly added node connects to node *i* with a probability $\frac{k_{i}+a}{\sum_{i}k_{i}+a}$ where *a* is an arbitrary constant) in blue (squares). Dashed lines correspond to the analytic mean for *W*(*x*), whereas the shaded areas indicate the standard deviation around the mean.

We have tested the behavior of *W*(*x*) by simulating *A* = 10000 attachment events on hierarchy *D* at year 2002, the results are shown in Fig 2. According to the plots, the measured *W*(*x*) remained within the standard deviation around the analytically calculated average for both purely random attachments (orange color), and linear preferential attachment with an additive constant.

When applying the above method for measuring preference in the empirical data, for every time step *t* (except for the last) we can measure the complementary cumulative distribution *Q*_*t*_(*x*), and count how many nodes having a property value at least as large as *x* have been selected by the given attachment mechanism between *t* and *t* + 1, denoted by *w*_*t*_(*x*). By aggregating their ratio in analogy with (1), we can define

$$\begin{array}{c} W_{\text{emp}}\left(x\right)=\sum_{t=1}^{t_{\text{max}}-1}\frac{w_{t}\left(x\right)}{Q_{t}\left(x\right)}. \end{array}$$ (8)

This can be compared to e.g., the mean and standard deviation of the random variable corresponding to the sum of the supposed *W*(*x*) under the assumption of independence from *x*, which according to (6 and 7) can be given as

$$\begin{array}{cc} \left\langle W_{\text{rand}}\left(x\right)\right\rangle & =\sum_{t=1}^{t_{\text{max}}-1}A_{t}, \end{array}$$ (9)

$$\begin{array}{cc} \sigma (W_{\text{rand}}\left(x\right)) & ={\left[\sum_{t=1}^{t_{\text{max}}-1}\frac{A_{t}\left(1-Q_{t}\left(x\right)\right)}{Q_{t}\left(x\right)}\right]}^{\frac{1}{2}}, \end{array}$$ (10)

where *A*_*t*_ denotes the number of attachment events between time steps *t* and *t* + 1.

#### Detachment events

An important difference between the addition of new links and link deletion events is that in the latter case, the natural assumption for the random choice (independent of any node property) is choosing a link uniformly at random from all existing links. The different nature of the two processes can be already seen in the domains over which they are defined, namely that attachment can occur between any pair of nodes, whereas detachment events can take place only over already existing links. A noteworthy consequence of this is that the degree of any node taking part in a detachment event is at least one before the event, which is not necessarily the case for attachment events.

When assuming a uniform random choice between the existing links, high degree nodes appear to be involved in the link deletion events with higher probability compared to low degree nodes simply because they have a higher number of connections. To take this into account, we have to redefine the formula of *Q*(*x*) for detachment events. First let us consider the case, where we are interested in whether some property of a node *x* has an effect on the likelihood that an out link is detached from it (also meaning that from the point of view of the deleted link, this node plays the role of source node). If we choose at random from all possible links, the probability that we pick an out link from a node with out degree *k*_out_ is given by *k*_out_ *p*(*k*_out_)/ 〈*k*_out_〉 (also referred to as access degree), where *p*(*k*_out_) denotes the out degree distribution, and 〈*k*_out_〉 is the average out degree (which is the same as the average in degree). Combining the previous arguments with the law of total probability, the probability distribution for property *x* on the source node of randomly selected links can be written as

$$\begin{array}{c} p_{\text{out}}\left(x\right)=\frac{1}{\left\langle k_{\text{out}}\right\rangle }\sum_{k_{\text{out}}}p\left(x|k_{\text{out}}\right)k_{\text{out}}p\left(k_{\text{out}}\right), \end{array}$$ (11)

where *p*(*x* ∣ *k*_out_) denotes the conditional probability that the property value is *x*, given that the out degree of the node is *k*_out_. Note that in the special case of a sharply peaked degree distribution around a characteristic value *k*_out_ ≈ 〈*k*_out_〉, the above expression for *p*_out_(*x*) coincides with the analogous formula of *p*(*x*) for attachment events. Based on *p*_out_(*x*), the complementary cumulative distribution *Q*_out_(*x*) can be calculated as usual,

$$\begin{array}{c} Q_{\text{out}}\left(x\right)=\sum_{x^{\prime }\geq x}p_{\text{out}}\left(x^{\prime }\right). \end{array}$$ (12)

If in contrast to out links, we are interested in the deletion of incoming links and the possible effect on the likelihood of such events by some node property *x*, we can formulate analogous formulas to the above using the in degree distribution *p*(*k*_in_). In this case the probability distribution for property *x* on the target node of randomly selected links can be written as

$$\begin{array}{c} p_{\text{in}}\left(x\right)=\frac{1}{\left\langle k_{\text{in}}\right\rangle }\sum_{k_{\text{in}}}p\left(x|k_{\text{in}}\right)k_{\text{in}}p\left(k_{\text{in}}\right), \end{array}$$ (13)

where *p*(*x* ∣ *k*_in_) denotes the conditional probability that the property value is *x*, given that the in degree of the node is *k*_in_, and the corresponding complementary cumulative distribution is given by

$$\begin{array}{c} Q_{\text{in}}\left(x\right)=\sum_{x^{\prime }\geq x}p_{\text{in}}\left(x^{\prime }\right). \end{array}$$ (14)

Otherwise, the analysis for the link deletion events is the same as in case of the attachment events: We can calculate *Q*_*t*_(*x*) using either (12) or (14), and by plugging the result together with the observed *w*_*t*_(*x*) into (8) we obtain *W*_emp_(*x*). To decide whether we can speak about a possible preference or anti-preference with respect to the chosen property, *W*_emp_(*x*) has to be compared to the *W*(*x*) expected based on neutral behaviour, calculated using (9 and 10).

## Results

We applied the methodology outlined in the previous section to study the time evolution of the hierarchies listed in Table 1 where the system size exceeds 1000 nodes during the whole recorded time period, corresponding to hierarchies A, B, C, D, E, G, and N. Before actually showing the results, first we need to specify the different possible attachment and detachment event types. In terms of the changing links we have two large categories: added (new) links and deleted links. When examining the endpoints of added links, both the source and the target can be either an already existing (old) node, or a new node, thus, there are altogether 4 types of added links. The case of deleted links is much simpler in this respect, as both endpoints must correspond to old nodes. Therefore, there are in total 5 different possibilities for changes in the connections. However, when examining the possible effect of a given node property on the likelihood that the node is going to take part in an attachment/detachment event, we also have to specify whether the node is the source or the target of the involved link. Thus, for any node property of interest we can examine 10 different scenarios over the time evolution of the hierarchies. Naturally, when interested in the possible effect of a node property of an old node, the value of the property is always measured before the link change event (e.g., if the change occurs between time steps *t* and *t* + 1, then it is recorded at *t*), whereas for new nodes we can only measure their properties at the time point of their appearance (i.e., at *t* + 1 for link change events between *t* and *t* + 1). We list the yearly frequencies of the different event types for the studied hierarchies in Tables A-G in S1 Text.

**Fig 3:**
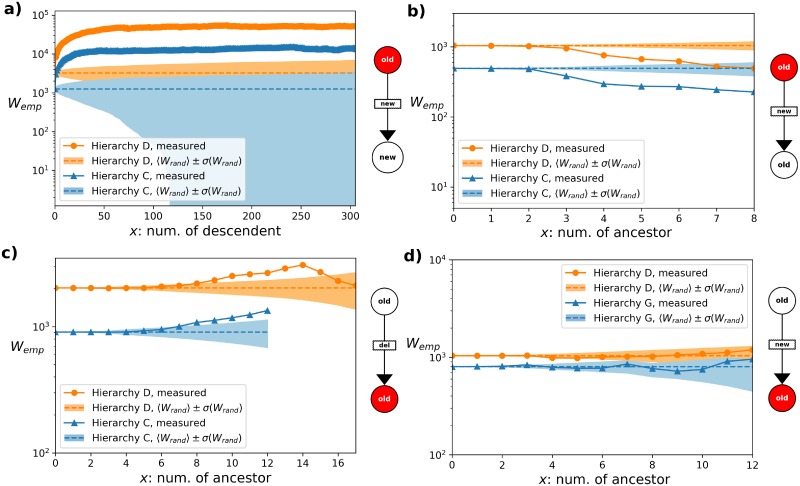
Measuring preference in attachment and detachment events. In each panel we compare *W*_emp_(*x*) defined in (8) to the mean and standard deviation of *W*(*x*) for random events, given in (9 and 10) and indicated by dashed lines in shaded areas. The pictograms beside the panels show the type of the studied attachment/detachment events and highlight in red whether the given property *x* was measured on the source or on the target of the links involved in the events. a) Results for the total number of descendants of source nodes in attachments of new links pointing from old nodes to new nodes in hierarchies D (orange) and C (blue). b) *W*_emp_(*x*) for the number of ancestors of source nodes on new links appearing between old nodes, measured in hierarchies D (orange) and C (blue). c) The same plots when *x* is equal to the number of ancestors of the target nodes in link deletion events for hierarchies D (orange) and C (blue). d) *W*_emp_(*x*) in case *x* is corresponding to the number of ancestors of the target node in attachment of new links between old nodes.

In our studies we focused on the following properties: number of children (out degree), number of parents (in degree), total number of descendants, total number of ancestors. As an illustration, in Fig 3. we show parts of the results obtained for hierarchies D, C and G. In Fig 3a the *W*_emp_(*x*) is plotted for hierarchies C and D, obtained from events where a new link pointing to a new node is attached to an old node, and *x* is corresponding to the total number of descendants of the source node. The curves indicate strong preference for large values of *x*, as they clearly exceed *W*_rand_(*x*) + σ (*W*_rand_(*x*)) by an order of magnitude. Interestingly somewhat the opposite can be seen in Fig 3b, showing the results for the same hierarchies in case of insertion of new links between pairs of already existing nodes, where *x* is corresponding to the total number of ancestors of the source node. The fact that *W*_emp_(*x*) is way below *W*_rand_(*x*) − σ (*W*_rand_(*x*)) indicates that the probability for the attachment of an incoming link to a node with higher number of ancestors is lower than what we would expect at random. In Fig 3c we considered link deletion events, and according to the results *W*_emp_(*x*) shows a non-monotonous behaviour as a function of *x* for the total number of ancestors of the target node in case of hierarchy D and a weak preference in case of hierarchy C. The peak in *W*_emp_(*x*) for hierarchy D is suggesting that there is a preferred value of *x*, where the likelihood of the node taking part in the given type of detachment event is maximal. Finally, in Fig 3d we show the results for the insertion of new links between old nodes (similarly to Fig 3b), but this time we depict *W*_emp_(*x*) for the total number of ancestors of the target node in case of hierarchies D and G. According to the results *W*_emp_(*x*) runs within the range of the standard deviation around 〈*W*_rand_(*x*)〉, thus, this type of attachment does not show any preference with respect to the number of ancestors of the target node.

Similar plots for the rest of the attachment/detachment types and for the other hierarchies are given in S1 Text. Based on the seen behaviour of *W*_emp_(*x*) we can categorise the observed behaviour as follows:

- Strong indication of preference (s+): *W*_emp_(*x*) shows a monotonous increasing behaviour, and exceeds 〈*W*_rand_(*x*)〉 + σ (*W*_rand_(*x*)) by a large amount, (as e.g., in case of Fig 3a).
- Weak indication of preference (w+): *W*_emp_(*x*) shows a monotonous increasing behaviour, exceeds 〈*W*_rand_(*x*)〉 + σ (*W*_rand_(*x*)), but only by a small amount.
- Strong indication of no preference (s0): *W*_emp_(*x*) remains within the standard deviation around 〈*W*(*x*)〉, (as e.g., in case of Fig 3d).
- Weak indication of anti-preference (w-): *W*_emp_(*x*) shows a monotonous decreasing behaviour, and falls under 〈*W*_rand_(*x*)〉 − σ (*W*_rand_(*x*)), by a small amount.
- Strong indication of anti-preference (s-): *W*_emp_(*x*) shows a monotonous decreasing behaviour, and falls under 〈*W*_rand_(*x*)〉 − σ (*W*_rand_(*x*)), by a larger amount, (as e.g., in case of Fig 3b)
- Indication of preference with a peak (p+): *W*_emp_(*x*) shows a non-monotonous behaviour, and has a maximum exceeding 〈*W*_rand_(*x*)〉 + σ (*W*_rand_(*x*)) by a significant amount, (as e.g., in case of Fig 3c).
- Indication of anti-preference with a peak (p-): *W*_emp_(*x*) shows a non-monotonous behaviour, and has a minimum falling under 〈*W*_rand_(*x*)〉 − σ (*W*_rand_(*x*)) by a significant amount.
- Insufficient statistics (i.s): in a number of cases it is not possible to draw a conclusion based on the empirical data. This may be due to the fact that the given type of attachment/detachment occurs rarely, or because that the distribution of the given node property is extremely narrow, resulting in a very limited range for *x*.

In Table 2 we give a summary overview of the results for the largest hierarchy (corresponding to hierarchy D), where the table is organised as follows: rows are corresponding to the 4 studied node properties, measured either on the source node (top 4 rows) or the target node (bottom 4 rows) of the changing links, and the table columns indicate the attachment/detachment types. In each cell we provide the category of the observed behaviour based on the corresponding plot. For example, the 3^rd^ cell in the 3^rd^ row is based on the orange curve in Fig 3a, the 4^th^ cell in the 4^th^ row is connected to Fig 3b, the 4^th^ cell in the last row is corresponding to Fig 3d, etc.

**Table 2:** Summary of the results for hierarchy D. The columns of the table correspond to the studied different link types, and the rows indicate the studied node property on either the source (top 4 rows) or the target (bottom 4 rows). The 3^rd^, 4^th^ and 5^th^ columns correspond to impossible link types, therefore, are left empty. The entries in the cells correspond to the following abbreviations: ‘s+’, ‘s0’ and ‘s-’ for strong indication of preference, no preference and anti-preference, ‘p+’ and ‘p-’ for indication of preference or anti-preference with a peak, and ‘i.s’ for insufficient statistics.

D		link: add				link: del			
		source: new		source: old		source: new		source: old	
		target: new	target: old	target: new	target: old	target: new	target: old	target: new	target: old
source	child.	s+	s+	s+	s+				s+
	par.	i.s.	i.s.	s−	i.s.				i.s.
	desc.	p−	s+	s+	s+				p+
	anc.	s0	s−	s−	s−				s0
target	child.	s−	i.s.	s−	s0				s0
	par.	i.s.	i.s.	s+	s0				s+
	desc.	s−	i.s.	s−	s0				s0
	anc.	s+	s0	s+	s0				p+

The overall pattern of the different preference types in Table 2 is highly non-trivial. E.g., all possible link change event types show preference with respect to the number of children of the source node (first row in Table 2), and all except for two (addition of new links between new nodes and deletion of old links) show anti-preference with respect to the total number of ancestors of the source node (4^th^ row in Table 2). Interestingly, in the 3^rd^ row of Table 2 (corresponding to the number of descendants of the source node) both preference and anti-preference is occurring among the cells corresponding to the different link change types. Seemingly the properties of the target nodes (bottom 4 rows) have a smaller effect compared to the properties of the source nodes (top 4 rows), indicated by the higher number of cells falling into the category of evidence for no-preference (s0). Nevertheless, preference with respect to the number of parents and number of ancestors, and anti-preference with respect to the number of children and number of descendants can be seen for a couple of the link change types.

Summary tables analogous to Table 2 for the other hierarchies are listed in the S1 Text. In order to be able to draw conclusions on the general features of the evolution of the studied hierarchies, we also provide an aggregated table with the same cell structure, in which the contribution from the individual tables were averaged in a simple manner, as shown in Table 3. According to that we can make the following observations about the presence of preference or anti-preference with respect to the different node properties during the growth and restructuring of the studied hierarchies:

- We can see strong signs of preference with respect to the number of children of the source node for both the addition of new links pointing from old nodes to new ones, and for the deletion of already existing links between old nodes.
- These link change events together with the addition of new links between already existing links clearly show preference with respect to the total number of descendants of the source node as well.
- All possible link change types show anti-preference with respect to the total number of ancestors of the source node. This effect is strong in case of addition of new links with an old source node, and for adding new links pointing from new nodes to old ones, whereas can be considered somewhat less pronounced for new links between two new nodes, and relatively weak for link deletions.
- We can see both preference, neutral behaviour and anti-preference with respect to the total number of ancestors of the target node: the addition of new links pointing to new nodes and link deletions seem to display a weak preference, the addition of new links between old nodes displays neutral behaviour, whereas in case of the addition of new links pointing from new nodes to old ones, we can observe a weak anti-preference.
- The attachment/detachment processes seem to be more influenced by the properties of the source node of the changing links, compared to the influence of the properties of the target nodes. This is supported by the fact that the top 4 row in Table 3 contains much higher number of cells with values (other than ‘i.s.’), and the magnitude of these is larger on average compared to cells in the bottom 4 rows.

**Table 3:** Aggregated summary results. Based on Table 2. and Tables H-N in S1 Text, the contribution to a given cell is counted according to ‘s+’ = 1, ‘w+’ = ’p+’ = 0.5, ‘s0 = 0’, ‘w–’ = ’p–’ = -0.5, ‘s–’ = -1, and the obtained sum is divided by the number of tables contributing to the given cell. Aggregated cells become ‘i.s’ if more than 3 out of the 7 tables has ‘i.s.’ as well.

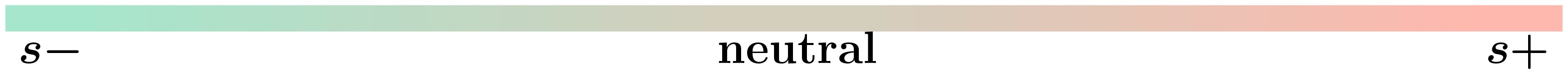									
Σ		link: add				link: del			
		source: new		source: old		source: new		source: old	
		target: new	target: old	target: new	target: old	target: new	target: old	target: new	target: old
source	child.	i.s.	i.s.	1.0,	i.s.				0.67,
	par.	i.s.	i.s.	i.s.	i.s.				i.s.
	desc.	i.s.	i.s.	0.89,	0.83,				0.61,
	asc.	-0.44,	-0.75,	-0.90,	-0.78,				-0.28,
target	child.	i.s.	i.s.	i.s.	i.s.				i.s.
	par.	i.s.	i.s.	i.s.	i.s.				i.s.
	desc.	i.s.	i.s.	i.s.	i.s.				i.s.
	asc.	0.24,	-0.11,	0.29,	0.0,				0.29,

An important further point to note is that the different hierarchies showed consistency in the sense that both preference and anti-preference was never observed simultaneously when comparing the same cells across the different summary tables.

## Discussion

We studied the change mechanisms of time evolving hierarchies between the PubMed MeSH terms using statistical methods. Although previous research has already shown interesting results regarding the growth of these networks [49–52], an important conclusion we can make based on our analysis is that deletion events and rewiring between already existing parts of the system are equally important in shaping the form of these hierarchies. This is supported by Tables A-G in S1 Text, according to which the number of deleted links together with the number of new links between already existing nodes under one time step is usually of the same magnitude as the number of new links connected to newly appearing nodes.

The main focus of our studies was on measuring preference during attachment and detachment events with respect to four different node properties characterising the hierarchy members. By setting up a general framework for this sort of analysis we could show that the likelihood for nodes to take part in restructuring events can be effected by their properties under quite a number of different circumstances. We found that when new links appear pointing from already existing nodes to newly appearing ones, the nodes with larger number of children (larger out degree) are chosen as source nodes for this type of links with significantly larger probabilities compared to uniform random choice. This effect is analogous to the preferential attachment rule of the Barabási–Albert network model [43], which was also observed empirically in different growing network systems [44–47]. However, in our case a larger number of children also increases the likelihood of loosing an out link (corresponding to a link deletion event).

Another property for which we observed similar behaviour is the total number of descendants, where in addition to the above two effects we could also detect preference during the addition of a new links pointing to other already existing nodes. In parallel, we observed anti-preference with respect to the number of ancestors of the source node for all possible link change types. Interestingly, if the node acts as the target of the changing link, we can observe both preference and anti-preference with respect to the number of ancestors for the different link change types. Since the number of descendants and the number of ancestors are defined only in case of hierarchies, the related results have no previously observed analogy in general time dependent networks.

From the practical point of view, the above findings also imply that based on the topological and hierarchical properties of the MeSH terms, it is possible to locate nodes in the present state of a MeSH hierarchy for which the likelihood of taking part in link change events in the future is higher compared to other nodes in the system. For the larger hierarchies, this sort of ‘flagging’ of the MeSH terms might help the curators carrying out the yearly update by providing candidates who’s neighborhood should be checked. However, it is also quite plausible that the inclusion of further properties not included in the present study (such as e.g., the frequency of the queries by the users) may turn out to be vital in further studies along this direction.

Finally, we note that according to Table 2 and Tables H-N in S1 Text one can observe a mild variance across the different hierarchies in terms of whether a given link change type displays some sort of preference with respect to a given property, or we see a neutral behaviour (or insufficient statistics) instead. Nevertheless, the results across the different hierarchies are consistent in the sense that we cannot observe both preference in case of one hierarchy, and anti-preference in case of another hierarchy for the same link change type and node property. This consistency is encouraging from the point of view of further research focusing on building network models for time evolving hierarchies. In addition, we note that although the empirical studies in this work are restricted to the networks between MeSH terms, it is quite plausible that a part of these features are more universal and occur in time evolution of networks with a hierarchical structure in general.

## Conclusion

In summary, our findings show that the growth and rewiring of the examined hierarchies are governed by non-trivial preference in the attachment mechanisms of the links. According to our results, the attachment is non-uniform with respect to multiple different topological and hierarchical node properties, and among the different possible link change scenarios we could observe both preferential and anti-preferential attachments, depending on the given node property of interest. These facts indicate that time evolution of these systems is far more complex compared to simple preferential attachment models, providing very interesting future challenges for modelling and further statistical analysis.

## Supporting information

S1 Text Supporting information. (PDF)
