# Supplementary material for: Time evolution of the hierarchical networks between PubMed MeSH terms
Source: PLoS One. 2019 Aug 12;14(8):e0220648. doi: 10.1371/journal.pone.0220648 (PMC6690519; doi:10.1371/journal.pone.0220648)
Supplement: S1 Text — (PDF) [file pone.0220648.s001.pdf]

Time evolution of the hierarchical networks between PubMed MeSH terms

Supporting Information

July 25, 2019

S1 Basic properties of the MeSH hierarchies

Owing to the yearly updates, the MeSH hierarchies evolve in time, displaying great number of structural changes that affect their topology both on the level of nodes and on the level of links. We studied the number of different annual change event types for each hierarchy with the corresponding results given in A-G Tables.

year	size	num. of del. nodes	num. of add. nodes	num. of del. links	num. of add. links			
					$o \to o$	$o \to n$	$n \to o$	$n \to n$
2002	1350	0	40	29	11	38	25	7
2003	1390	1	34	49	27	39	38	4
2004	1423	1	11	5	6	17	1	1
2005	1433	3	38	39	12	41	20	8
2006	1468	5	29	10	6	28	2	9
2007	1492	1	38	35	36	34	5	11
2008	1529	4	33	36	14	38	30	4
2009	1558	0	58	0	4	24	10	47
2010	1616	1	32	3	0	40	3	2
2011	1647	1	30	5	10	32	6	1
2012	1676	1	9	1	1	10	0	0
2013	1684	1	20	1	0	12	1	10
2014	1703	0	61	23	9	58	23	16
2015	1764	1	17	27	11	27	3	3
2016	1780	0	39	9	5	41	5	0
2017	1819	0	7	0	2	11	0	0

Table A. Number of different annual change event types in hierarchy A. The 1st column displays the year of observation with the corresponding network size given in the 2nd column. The 3rd and 4th columns correspond to the number of deleted and added nodes within that particular year. The 5th column displays the number of deleted links while the 6th, 7th, 8th, 9th columns correspond to the number of link addition between old nodes, between old sources and new targets, between new sources and old targets, and between new nodes.

year	size	num. of del. nodes	num. of add. nodes	num. of del. links	num. of add. links			
					$o \to o$	$o \to n$	$n \to o$	$n \to n$
2002	2252	9	722	85	37	622	46	104
2003	2965	19	243	156	53	252	61	38
2004	3189	9	171	48	22	157	27	17
2005	3351	12	96	39	5	60	22	37
2006	3435	15	62	51	18	62	12	5
2007	3482	0	16	3	5	16	1	1
2008	3498	1	57	7	4	63	2	7
2009	3554	40	57	81	3	26	31	42
2010	3571	2	62	19	9	57	8	12
2011	3631	0	25	7	0	29	7	2
2012	3656	3	4	5	2	4	0	0
2013	3657	0	13	3	0	8	2	5
2014	3670	1	14	2	0	13	0	1
2015	3683	4	27	76	26	24	37	4
2016	3706	3	68	54	7	60	57	11
2017	3771	5	49	180	78	36	98	13

Table B. Number of different annual change event types in hierarchy B. The arrangement of the table is the same as in case of A Table: 1st column is the year, 2nd column is the size, 3rd column is for the deleted nodes, 4th column is for the added nodes, 5th column is for the deleted links, and the columns from 6th to 9th display the different types of added links.

year	size	num. of del. nodes	num. of add. nodes	num. of del. links	num. of add. links			
					$o \to o$	$o \to n$	$n \to o$	$n \to n$
2002	3975	3	43	28	20	58	39	3
2003	4015	5	44	99	59	58	25	4
2004	4054	5	61	82	50	68	22	6
2005	4110	11	65	79	31	77	47	10
2006	4164	5	71	95	73	95	24	6
2007	4230	34	73	153	72	94	18	11
2008	4269	4	59	45	23	70	19	11
2009	4324	6	91	43	22	161	30	10
2010	4409	6	92	45	36	139	18	7
2011	4495	0	83	16	19	125	21	13
2012	4578	4	23	9	6	32	0	1
2013	4597	4	28	35	15	34	14	3
2014	4621	3	46	21	4	61	9	6
2015	4664	0	23	103	26	28	3	0
2016	4687	0	72	21	19	91	30	6
2017	4759	0	40	32	17	61	30	0

Table C. Number of different annual change event types in hierarchy C. The arrangement of the table is the same as in case of A Table: 1st column is the year, 2nd column is the size, 3rd column is for the deleted nodes, 4th column is for the added nodes, 5th column is for the deleted links, and columns from 6th to 9th display the different types of added links.

year	size	num. of del. nodes	num. of add. nodes	num. of del. links	num. of add. links			
					$o \to o$	$o \to n$	$n \to o$	$n \to n$
2002	6902	10	251	166	74	225	86	148
2003	7143	56	269	329	111	235	181	106
2004	7356	23	142	109	76	119	76	55
2005	7475	34	700	389	224	651	154	330
2006	8141	5	269	141	95	263	43	113
2007	8405	6	219	72	78	200	51	87
2008	8618	1	103	21	12	88	13	53
2009	8720	5	101	43	24	99	17	53
2010	8816	7	166	64	26	152	64	63
2011	8975	14	115	64	22	124	33	38
2012	9076	23	108	182	113	100	74	53
2013	9161	3	122	76	34	124	48	40
2014	9280	1	74	18	17	65	10	30
2015	9353	1	165	101	20	224	13	42
2016	9517	1	234	52	56	267	72	77
2017	9750	2	186	198	56	279	39	36

Table D. Number of different annual change event types in hierarchy D. The arrangement of the table is the same as in case of A Table: 1st column is the year, 2nd column is the size, 3rd column is for the deleted nodes, 4th column is for the added nodes, 5th column is for the deleted links, and columns from 6th to 9th display the different types of added links.

year	size	num. of del. nodes	num. of add. nodes	num. of del. links	num. of add. links			
					$o \to o$	$o \to n$	$n \to o$	$n \to n$
2002	2040	5	57	19	6	60	12	6
2003	2092	0	28	5	7	28	4	3
2004	2120	3	59	33	22	43	23	26
2005	2176	5	29	32	22	33	6	1
2006	2200	3	43	12	6	47	10	6
2007	2240	4	31	36	15	35	12	3
2008	2267	5	113	30	16	79	32	49
2009	2375	0	70	25	28	78	6	5
2010	2445	5	113	39	18	133	39	8
2011	2553	6	83	63	57	103	14	6
2012	2630	1	74	8	2	79	15	7
2013	2703	4	28	15	6	28	5	2
2014	2727	0	34	23	13	37	1	0
2015	2761	3	60	71	14	62	14	10
2016	2818	4	51	20	11	62	1	1
2017	2865	1	60	9	9	64	9	10

Table E. Number of different annual change event types in hierarchy E. The arrangement of the table is the same as in case of A Table: 1st column is the year, 2nd column is the size, 3rd column is for the deleted nodes, 4th column is for the added nodes, 5th column is for the deleted links, and columns from 6th to 9th display the different types of added links.

year	size	num. of del. nodes	num. of add. nodes	num. of del. links	num. of add. links			
					$o \to o$	$o \to n$	$n \to o$	$n \to n$
2002	1803	4	112	111	52	50	83	78
2003	1911	32	84	159	31	50	94	47
2004	1963	17	48	84	41	46	33	9
2005	1994	19	52	79	27	54	26	5
2006	2027	8	64	69	21	57	59	11
2007	2083	3	38	23	24	39	4	4
2008	2118	632	247	1059	123	65	345	229
2009	1733	4	37	29	17	38	16	4
2010	1766	4	75	13	11	89	4	16
2011	1837	6	91	26	16	108	14	16
2012	1922	0	28	4	0	31	2	2
2013	1950	0	28	19	4	34	12	1
2014	1978	1	48	10	7	53	3	16
2015	2025	3	170	54	12	45	22	145
2016	2192	44	68	483	410	67	14	17
2017	2216	0	43	10	6	45	11	3

Table F. Number of different annual change event types in hierarchy G. The arrangement of the table is the same as in case of A Table: 1st column is the year, 2nd column is the size, 3rd column is for the deleted nodes, 4th column is for the added nodes, 5th column is for the deleted links, and columns from 6th to 9th display the different types of added links.

year	size	num. of del. nodes	num. of add. nodes	num. of del. links	num. of add. links			
					$o \to o$	$o \to n$	$n \to o$	$n \to n$
2002	1072	0	36	1	0	39	7	2
2003	1108	0	5	6	4	3	2	2
2004	1113	1	7	1	0	7	0	0
2005	1119	1	3	2	0	4	1	0
2006	1121	0	1	0	0	1	0	0
2007	1122	2	38	22	11	37	4	4
2008	1158	0	254	6	11	43	20	244
2009	1412	0	31	3	16	34	0	4
2010	1443	0	43	2	3	45	4	5
2011	1486	0	38	5	2	41	5	1
2012	1524	10	52	16	0	50	3	7
2013	1566	3	34	4	1	38	0	2
2014	1597	1	25	5	5	24	0	3
2015	1621	3	79	35	18	85	5	9
2016	1697	9	66	24	10	68	4	6
2017	1754	0	41	8	4	46	4	1

Table G. Number of different annual change event types in hierarchy N. The arrangement of the table is the same as in case of A Table: 1st column is the year, 2nd column is the size, 3rd column is for the deleted nodes, 4th column is for the added nodes, 5th column is for the deleted links, and columns from 6th to 9th display the different types of added links.

S2 The overall pattern of the different preference types

The observed different preference types among all possible link change scenarios are listed in H-N Tables, each of which corresponds to a different hierarchy.

A		link: add				link: del			
		source: new		source: old		source: new		source: old	
		target: new	target: old	target: new	target: old	target: new	target: old	target: new	target: old
source	child.	i.s.	i.s.	s+	i.s.				w+
	par.	i.s.	i.s.	i.s.	i.s.				i.s.
	desc.	i.s.	w+	p+	i.s.				p+
	anc.	i.s.	i.s.	s−	w−				i.s.
target	child.	i.s.	i.s.	i.s.	i.s.				i.s.
	par.	i.s.	i.s.	i.s.	i.s.				i.s.
	desc.	i.s.	i.s.	i.s.	i.s.				i.s.
	anc.	s0	s0	s0	i.s.				s0

Table H. Summary of the results for hierarchy A. The columns of the table display different link types, while the rows correspond to the examined node property on either the source (top 4 rows) or the target (bottom 4 rows). The 3rd, 4th and 5th columns correspond to forbidden link types highlighted in grey. Symbols inside the cells refer to the following abbreviations: 's+', 's0' and 's-' for indication of strong preference, no preference (neutrality) and strong anti-preference, 'w+' and 'w-' for weak preference and anti-preference, while 'p+' and 'p-' symbolize preference or anti-preference with a non-trivial peak, and 'i.s' for insufficient statistics.

B		link: add				link: del			
		source: new		source: old		source: new		source: old	
		target: new	target: old	target: new	target: old	target: new	target: old	target: new	target: old
source	child.	i.s.	i.s.	s+	i.s.				w+
	par.	i.s.	i.s.	i.s.	i.s.				i.s.
	desc.	i.s.	i.s.	s+	i.s.				w+
	anc.	w−	i.s.	p−	i.s.				w−
target	child.	i.s.	i.s.	i.s.	i.s.				i.s.
	par.	i.s.	i.s.	i.s.	i.s.				i.s.
	desc.	i.s.	i.s.	i.s.	i.s.				i.s.
	anc.	s0	s0	s0	i.s.				p+

Table I. Summary of the results for hierarchy B. The arrangement and abbreviations of the table are the same as in case of H Table.

C		link: add				link: del			
		source: new		source: old		source: new		source: old	
		target: new	target: old	target: new	target: old	target: new	target: old	target: new	target: old
source	child.	i.s.	i.s.	s+	s+				s0
	par.	i.s.	i.s.	i.s.	i.s.				i.s.
	desc.	i.s.	s+	s+	s+				s0
	anc.	s0	s−	s−	s−				s0
target	child.	i.s.	i.s.	i.s.	i.s.				i.s.
	par.	i.s.	i.s.	i.s.	i.s.				i.s.
	desc.	i.s.	i.s.	i.s.	i.s.				i.s.
	anc.	s0	s0	s+	s0				w+

Table J. Summary of the results for hierarchy C. The arrangement and abbreviations of the table are the same as in case of H Table.

D		link: add				link: del			
		source: new		source: old		source: new		source: old	
		target: new	target: old	target: new	target: old	target: new	target: old	target: new	target: old
source	child.	s+	s+	s+	s+				s+
	par.	i.s.	i.s.	w−	i.s.				i.s.
	desc.	p−	s+	s+	p+				p+
	anc.	s0	s−	s−	s−				s0
target	child.	i.s.	i.s.	s−	s0				s0
	par.	i.s.	i.s.	s+	s0				s+
	desc.	w−	i.s.	s−	s0				s0
	anc.	s+	s0	s+	s0				p+

Table K. Summary of the results for hierarchy D. The arrangement and abbreviations of the table are the same as in case of H Table.

E		link: add				link: del			
		source: new		source: old		source: new		source: old	
		target: new	target: old	target: new	target: old	target: new	target: old	target: new	target: old
source	child.	i.s.	i.s.	s+	i.s.				w+
	par.	i.s.	i.s.	i.s.	i.s.				i.s.
	desc.	i.s.	i.s.	s+	i.s.				w+
	anc.	w−	i.s.	s−	s−				s0
target	child.	i.s.	i.s.	i.s.	i.s.				i.s.
	par.	i.s.	i.s.	i.s.	i.s.				i.s.
	desc.	i.s.	i.s.	i.s.	i.s.				i.s.
	anc.	s0	i.s.	s0	s0				s0

Table L. Summary of the results for hierarchy E. The arrangement and abbreviations of the table are the same as in case of H Table.

G		link: add				link: del			
		source: new		source: old		source: new		source: old	
		target: new	target: old	target: new	target: old	target: new	target: old	target: new	target: old
source	child.	i.s.	i.s.	s+	s+				s+
	par.	i.s.	i.s.	i.s.	i.s.				i.s.
	desc.	i.s.	i.s.	p+	s+				s+
	anc.	w−	s−	p−	s−				s−
target	child.	i.s.	i.s.	i.s.	i.s.				w+
	par.	i.s.	i.s.	i.s.	i.s.				i.s.
	desc.	i.s.	i.s.	i.s.	i.s.				w+
	anc.	s0	w−	s0	s0				s0

Table M. Summary of the results for hierarchy G. The arrangement and abbreviations of the table are the same as in case of H Table.

N		link: add				link: del			
		source: new		source: old		source: new		source: old	
		target: new	target: old	target: new	target: old	target: new	target: old	target: new	target: old
source	child.	i.s.	i.s.	i.s.	i.s.				i.s.
	par.	i.s.	i.s.	i.s.	i.s.				i.s.
	desc.	i.s.	i.s.	i.s.	w+				i.s.
	anc.	w−	s0	s−	s0				s0
target	child.	i.s.	i.s.	i.s.	i.s.				i.s.
	par.	i.s.	i.s.	i.s.	i.s.				i.s.
	desc.	i.s.	i.s.	i.s.	i.s.				i.s.
	anc.	w+	s0	s0	s0				s0

Table N. Summary of the results for hierarchy N. The arrangement and abbreviations of the table are the same as in case of H Table.

S3 Measuring preference in the attachment and detachment events

The numerical results obtained for hierarchies A,B,C,D,E,G,N are depicted in A-K Figs. each of which is composed of several different panels. We display only those attachment/detachment types are in the sub figures where the statistics were found to be sufficient. In each panel $W_{\mathrm{emp}}(x)$ defined in Eq.(8) in the main paper as

$$W_{\mathrm{emp}}(x) = \sum_{t=1}^{t_{\max}-1} \frac{w_t(x)}{Q_t(x)} \tag{S1}$$

is compared to the predicted behavior of the mean and the standard deviation of $W(x)$ (given in Eqs. (9-10) in the main paper) for random events represented by dashed lines in shaded areas.

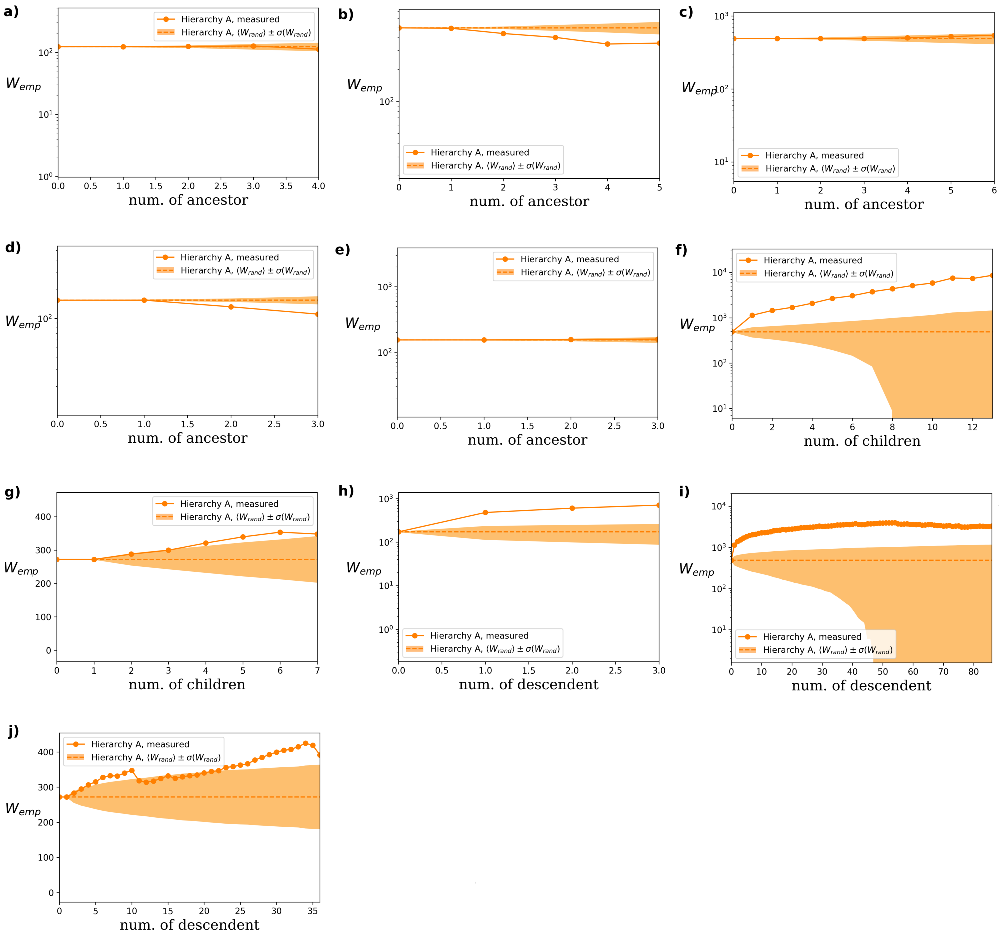

Fig A. Results for hierarchy A. a) Addition of new links between new nodes, on the horizontal axis we show the num. of ancestors of the target node. b) Addition of new links pointing from old to new nodes, on the horizontal axis we show the num. of ancestors of the source node. c) Addition of new links pointing from old to new nodes, on the horizontal axis we show the num. of ancestors of the target node. d) Addition of new links between old nodes, on the horizontal axis we show the num. of ancestors of the source node. e) Addition of new links between old nodes, on the horizontal axis we show the num. of ancestors of the target node. f) Addition of new links pointing from old to new nodes, on the horizontal axis we show the num. of children of the source node. g) Deletion of links between old nodes, on the horizontal axis we show the num. of children of the source node. h) Addition of new links pointing from new to old nodes, on the horizontal axis we show the num. of descendents of the source node. i) Addition of new links pointing from old to new nodes, on the horizontal axis we show the num. of descendents of the source node. j) Deletion of links between old nodes, on the horizontal axis we show the num. of descendents of the source node.

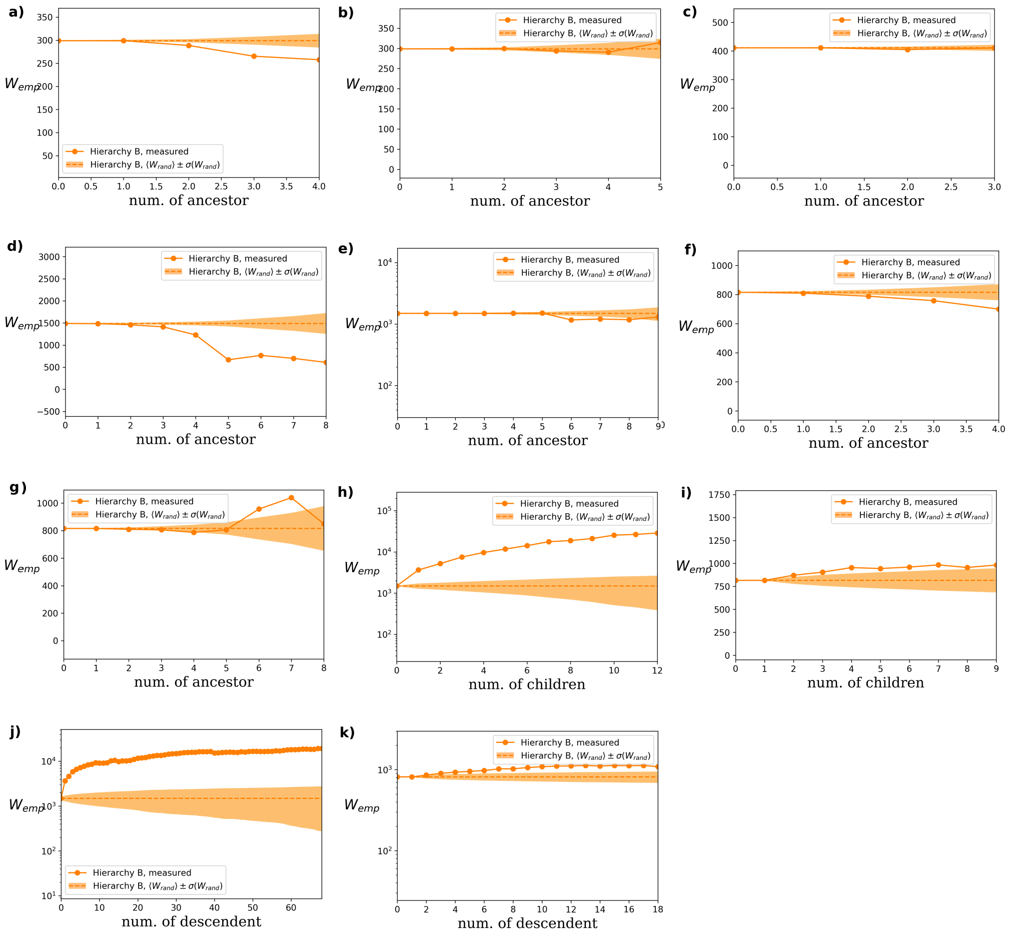

Fig B. Results for hierarchy B. a) Addition of new links between new nodes, on the horizontal axis we show the num. of ancestors of the source node. b) Addition of new links between new nodes, on the horizontal axis we show the num. of ancestors of the target node. c) Addition of new links pointing from new to old nodes, on the horizontal axis we show the num. of ancestors of the target node. d) Addition of new links pointing from old to new nodes, on the horizontal axis we show the num. of ancestors of the source node. e) Addition of new links pointing from old to new nodes, on the horizontal axis we show the num. of ancestors of the target node. f) Deletion of links between old nodes, on the horizontal axis we show the num. of ancestors of the source node. g) Deletion of links between old nodes, on the horizontal axis we show the num. of ancestors of the target node. h) Addition of new links pointing from old to new nodes, on the horizontal axis we show the num. of children of the source node. i) Deletion of links between old nodes, on the horizontal axis we show the num. of children of the source node. j) Addition of new links pointing from old to new nodes, on the horizontal axis we show the num. of descendents of the source node. k) Deletion of links between old nodes, on the horizontal axis we show the num. of descendents of the source node.

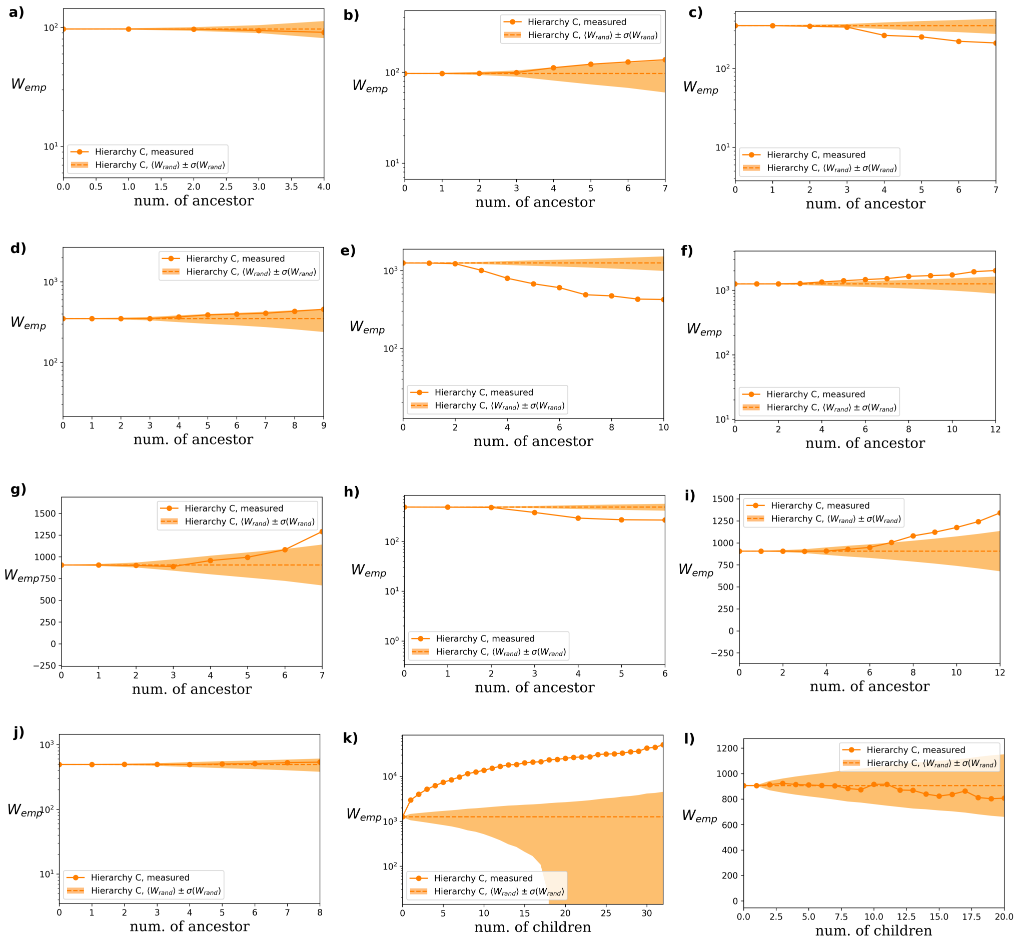

Fig C. Results for hierarchy C. a) Addition of new links between new nodes, on the horizontal axis we show the num. of ancestors of the source node. b) Addition of new links between new nodes, on the horizontal axis we show the num. of ancestors of the target node. c) Addition of new links pointing from new to old nodes, on the horizontal axis we show the num. of ancestors of the source node. d) Addition of new links pointing from new to old nodes, on the horizontal axis we show the num. of ancestors of the target node. e) Addition of new links pointing from old to new nodes, on the horizontal axis we show the num. of ancestors of the source node. f) Addition of new links pointing from old to new nodes, on the horizontal axis we show the num. of ancestors of the target node. g) Deletion of links between old nodes, on the horizontal axis we show the num. of ancestors of the source node. h) Addition of new links between old nodes, on the horizontal axis we show the num. of ancestors of the source node. i) Deletion of links between old nodes, on the horizontal axis we show the num. of ancestors of the target node. j) Addition of new links between old nodes, on the horizontal axis we show the num. of ancestors of the target node. k) Addition of new links pointing from old to new nodes, on the horizontal axis we show the num. of children of the source node. l) Deletion of links between old nodes, on the horizontal axis we show the num. of children of the source node.

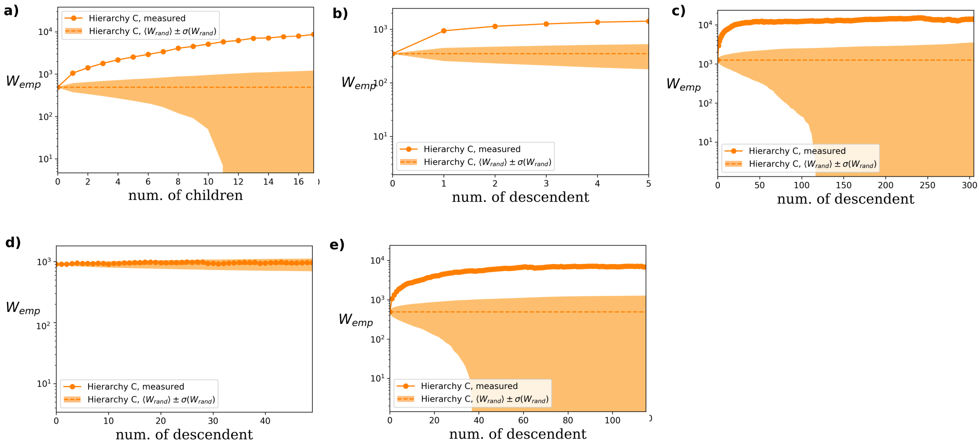

Fig D. Results for hierarchy C. a) Addition of new links between old nodes, on the horizontal axis we show the num. of children of the source node. b) Addition of new links pointing from new to old nodes, on the horizontal axis we show the num. of descendents of the source node. c) Addition of new links pointing from old to new nodes, on the horizontal axis we show the num. of descendents of the source node. d) Deletion of links between old nodes, on the horizontal axis we show the num. of descendents of the source node. e) Addition of new links between old nodes, on the horizontal axis we show the num. of descendents of the source node.

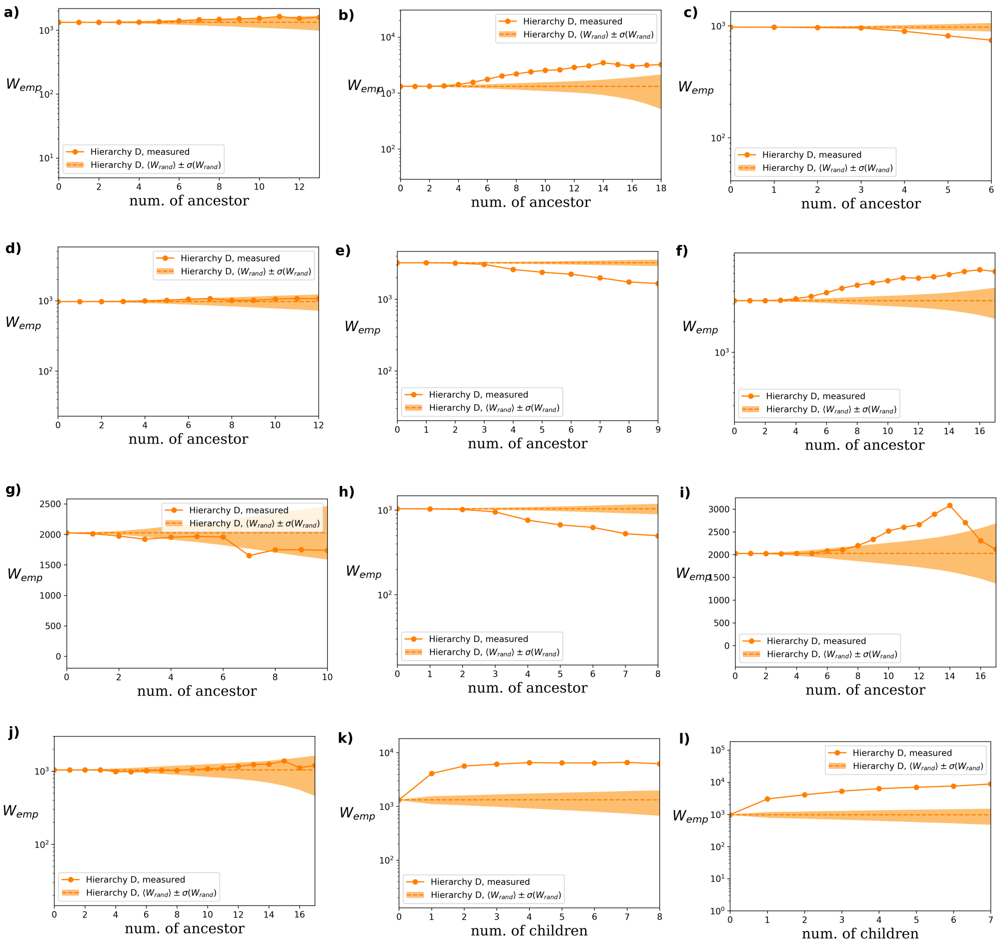

Fig E. Results for hierarchy D. a) Addition of new links between new nodes, on the horizontal axis we show the num. of ancestors of the source node. b) Addition of new links between new nodes, on the horizontal axis we show the num. of ancestors of the target node. c) Addition of new links pointing from new to old nodes, on the horizontal axis we show the num. of ancestors of the source node. d) Addition of new links pointing from new to old nodes, on the horizontal axis we show the num. of ancestors of the target node. e) Addition of new links pointing from old to new nodes, on the horizontal axis we show the num. of ancestors of the source node. f) Addition of new links pointing from old to new nodes, on the horizontal axis we show the num. of ancestors of the target node. g) Deletion of links between old nodes, on the horizontal axis we show the num. of ancestors of the source node. h) Addition of new links between old nodes, on the horizontal axis we show the num. of ancestors of the source node. i) Deletion of links between old nodes, on the horizontal axis we show the num. of ancestors of the target node. j) Addition of new links between old nodes, on the horizontal axis we show the num. of ancestors of the target node. k) Addition of new links between new nodes, on the horizontal axis we show the num. of children of the source node. l) Addition of new links pointing from new to old nodes, on the horizontal axis we show the num. of children of the source node.

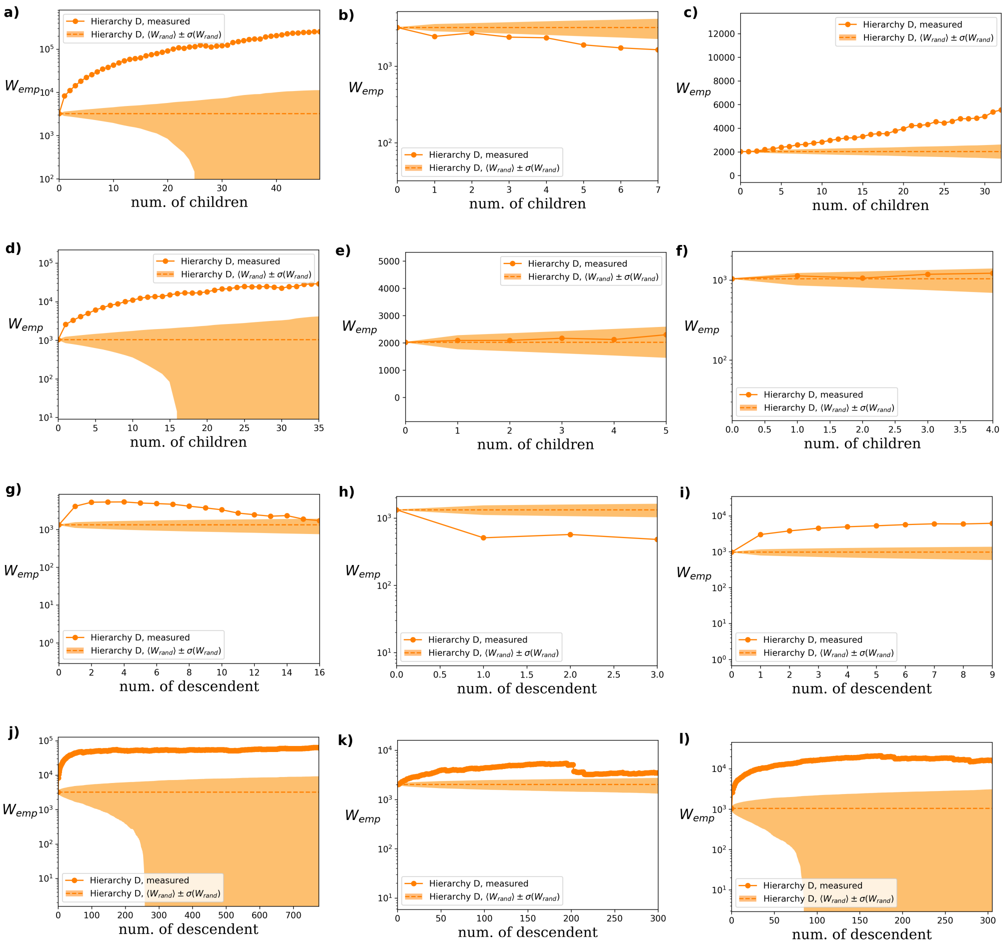

Fig F. Results for hierarchy D. a) Addition of new links pointing from old to new nodes, on the horizontal axis we show the num. of children of the source node. b) Addition of new links pointing from old to new nodes, on the horizontal axis we show the num. of children of the target node. c) Deletion of links between old nodes, on the horizontal axis we show the num. of children of the source node. d) Addition of new links between old nodes, on the horizontal axis we show the num. of children of the source node. e) Deletion of links between old nodes, on the horizontal axis we show the num. of children of the target node. f) Addition of new links between old nodes, on the horizontal axis we show the num. of children of the target node. g) Addition of new links between new nodes, on the horizontal axis we show the num. of descendents of the source node. h) Addition of new links between new nodes, on the horizontal axis we show the num. of descendents of the target node. i) Addition of new links pointing from new to old nodes, on the horizontal axis we show the num. of descendents of the source node. j) Addition of new links pointing from old to new nodes, on the horizontal axis we show the num. of descendents of the source node. k) Deletion of links between old nodes, on the horizontal axis we show the num. of descendents of the source node. l) Addition of new links between old nodes, on the horizontal axis we show the num. of descendents of the source node.

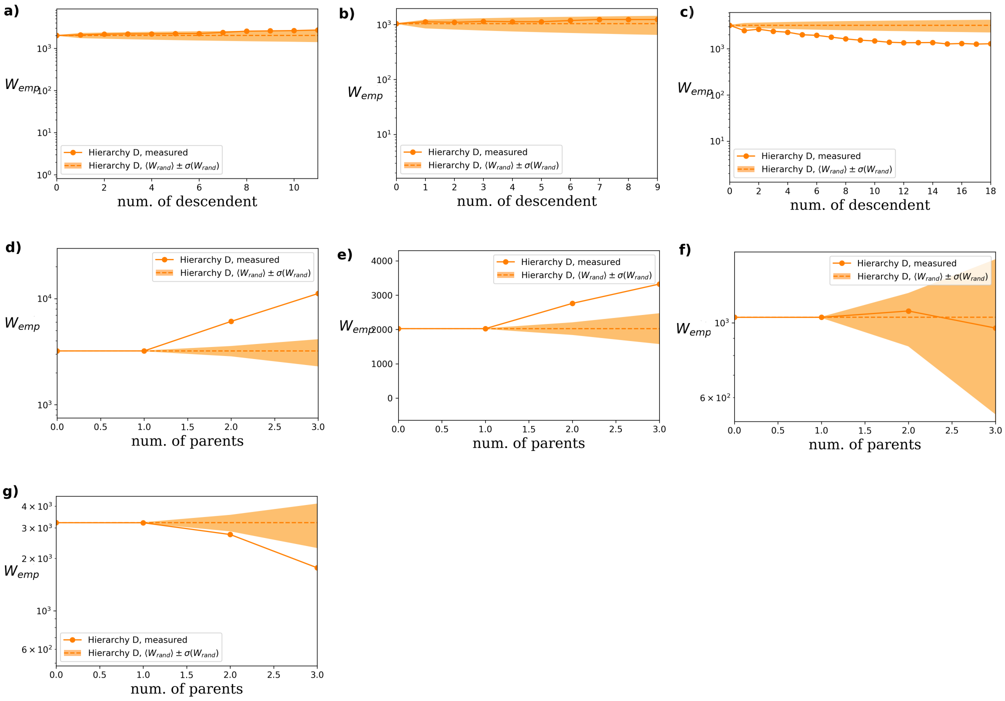

Fig G. Results for hierarchy D. a) Deletion of links between old nodes, on the horizontal axis we show the num. of descendents of the target node. b) Addition of new links between old nodes, on the horizontal axis we show the num. of descendents of the target node. c) Addition of new links pointing from old to new nodes, on the horizontal axis we show the num. of descendents of the target node. d) Addition of new links pointing from old to new nodes, on the horizontal axis we show the num. of parents of the target node. e) Deletion of links between old nodes, on the horizontal axis we show the num. of parents of the target node. f) Addition of links between old nodes, on the horizontal axis we show the num. of parents of the target node. g) Addition of new links pointing from old to new nodes, on the horizontal axis we show the num. of parents of the source node.

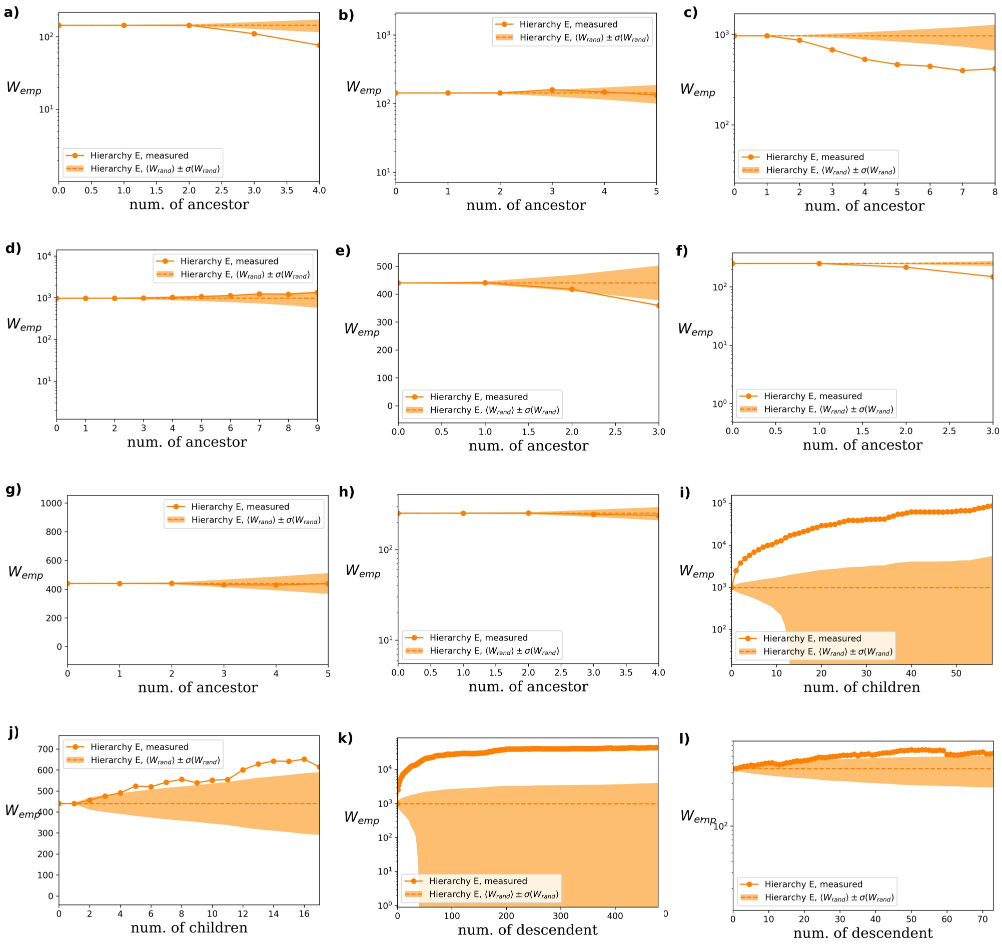

Fig H. Results for hierarchy E. a) Addition of new links between new nodes, on the horizontal axis we show the num. of ancestors of the source node. b) Addition of new links between new nodes, on the horizontal axis we show the num. of ancestors of the target node. c) Addition of new links pointing from old to new nodes, on the horizontal axis we show the num. of ancestors of the source node. d) Addition of new links pointing from old to new nodes, on the horizontal axis we show the num. of ancestors of the target node. e) Deletion of links between old nodes, on the horizontal axis we show the num. of ancestors of the source node. f) Addition of new between old nodes, on the horizontal axis we show the num. of ancestors of the source node. g) Deletion of links between old nodes, on the horizontal axis we show the num. of ancestors of the target node. h) Addition of new links between old nodes, on the horizontal axis we show the num. of ancestors of the target node. i) Addition of new links pointing from old to new nodes, on the horizontal axis we show the num. of children of the source node. j) Deletion of links between old nodes, on the horizontal axis we show the num. of children of the source node. k) Addition of new links pointing from old to new nodes, on the horizontal axis we show the num. of descendents of the source node. l) Deletion of links between old nodes, on the horizontal axis we show the num. of descendents of the source node.

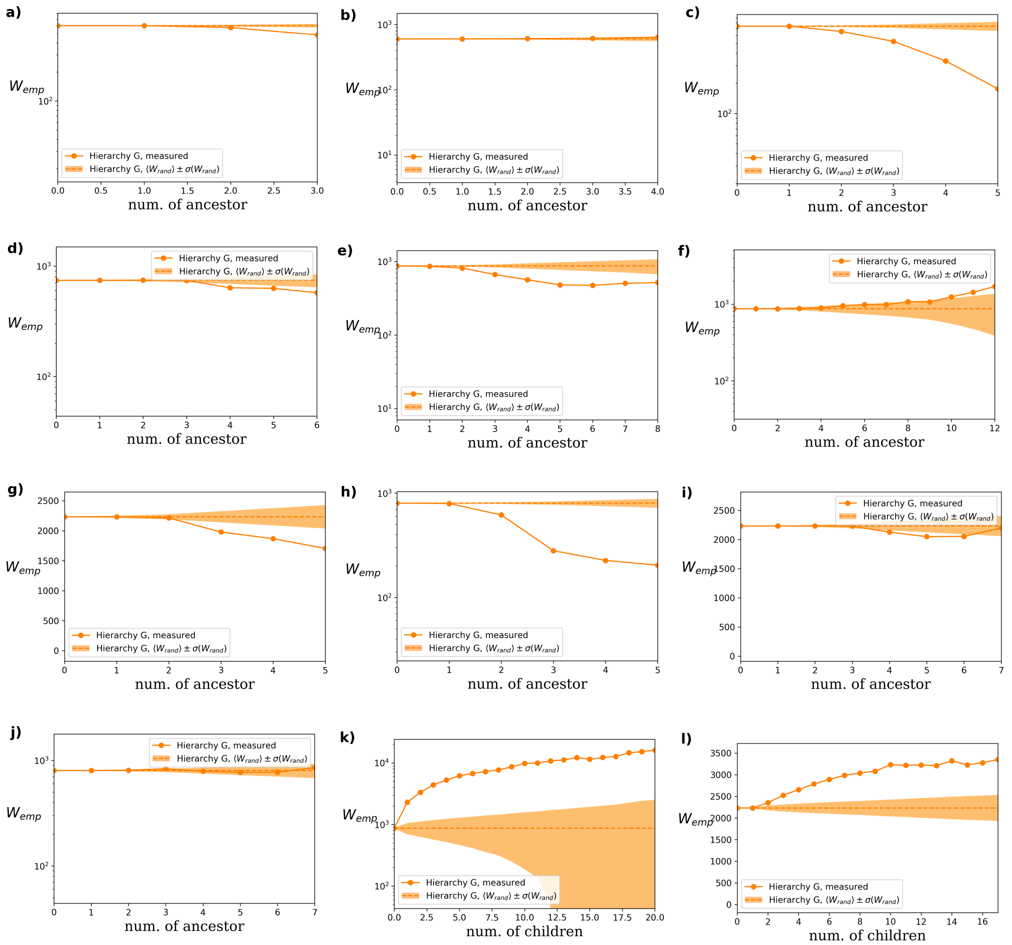

Fig I. Results for hierarchy G. a) Addition of new links between new nodes, on the horizontal axis we show the num. of ancestors of the source node. b) Addition of new links between new nodes, on the horizontal axis we show the num. of ancestors of the target node. c) Addition of new links pointing from new to old nodes, on the horizontal axis we show the num. of ancestors of the source node. d) Addition of new links pointing from new to old nodes, on the horizontal axis we show the num. of ancestors of the target node. e) Addition of new links pointing from old to new nodes, on the horizontal axis we show the num. of ancestors of the source node. f) Addition of new links pointing from old to new nodes, on the horizontal axis we show the num. of ancestors of the target node. g) Deletion of links between old nodes, on the horizontal axis we show the num. of ancestors of the source node. h) Addition of new links between old nodes, on the horizontal axis we show the num. of ancestors of the source node. i) Deletion of links between old nodes, on the horizontal axis we show the num. of ancestors of the target node. j) Addition of new links between old nodes, on the horizontal axis we show the num. of ancestors of the target node. k) Addition of new links pointing from old to new nodes, on the horizontal axis we show the num. of children of the source node. l) Deletion of links between old nodes, on the horizontal axis we show the num. of children of the source node.

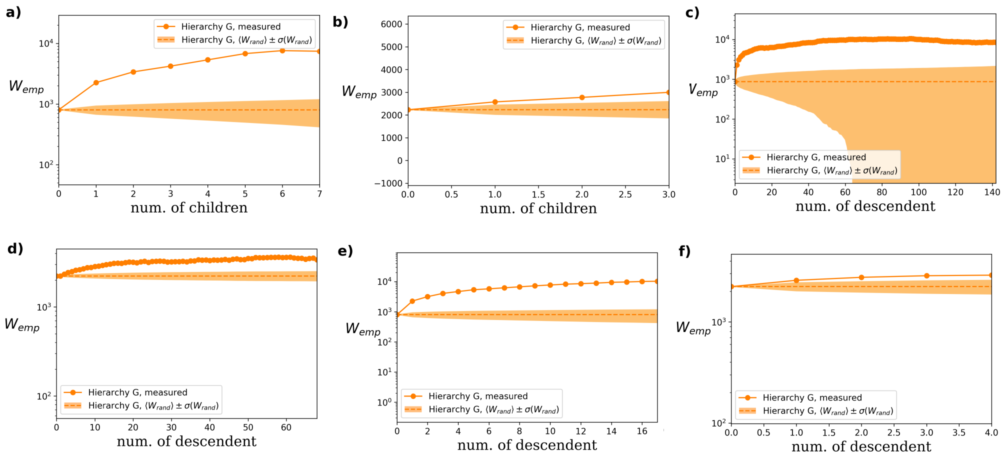

Fig J. Results for hierarchy G. a) Addition of new links between old nodes, on the horizontal axis we show the num. of children of the source node. b) Deletion of links between old nodes, on the horizontal axis we show the num. of children of the target node. c) Addition of new links pointing from old to new nodes, on the horizontal axis we show the num. of descendents of the source node. d) Deletion of links between old nodes, on the horizontal axis we show the num. of descendents of the source node. e) Addition of new links between old nodes, on the horizontal axis we show the num. of descendents of the source node. f) Deletion of links between old nodes, on the horizontal axis we show the num. of descendents of the target node.

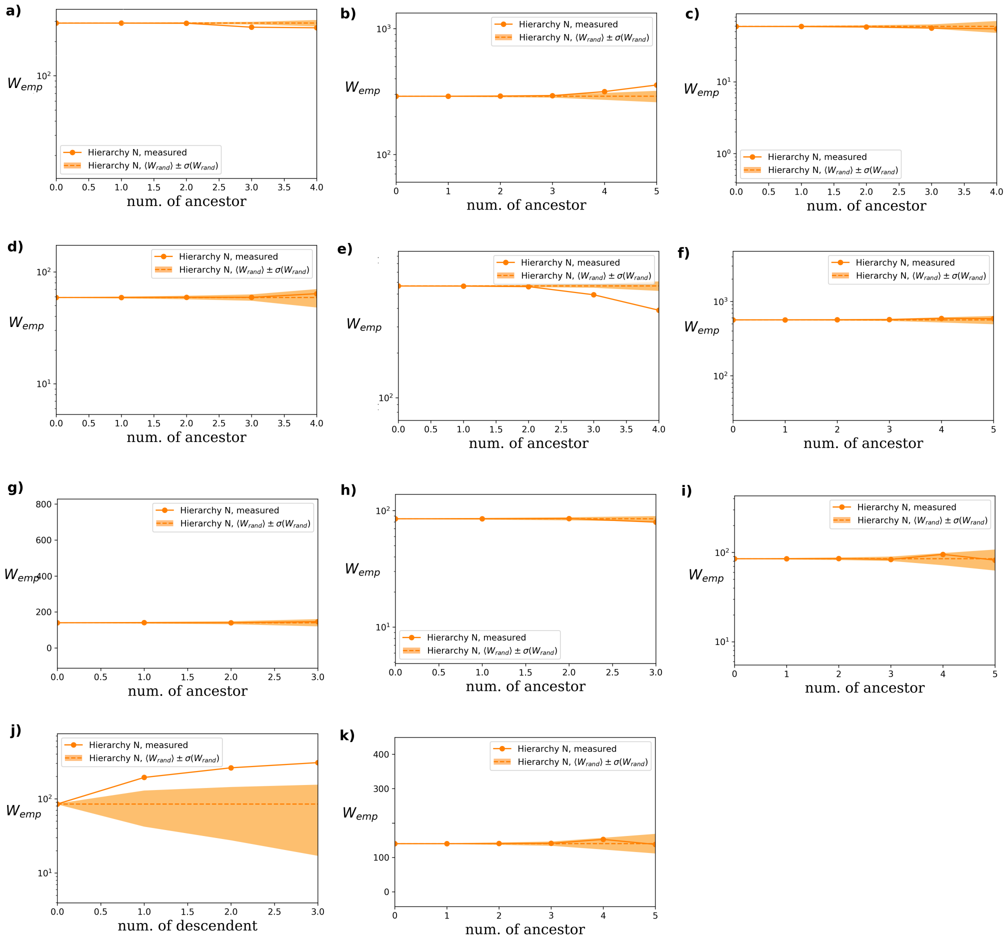

Fig K. Results for hierarchy N. a) Addition of new links between new nodes, on the horizontal axis we show the num. of ancestors of the source node. b) Addition of new links between new nodes, on the horizontal axis we show the num. of ancestors of the target node. c) Addition of new links pointing from new to old nodes, on the horizontal axis we show the num. of ancestors of the source node. d) Addition of new links pointing from new to old nodes, on the horizontal axis we show the num. of ancestors of the target node. e) Addition of new links pointing from old to new nodes, on the horizontal axis we show the num. of ancestors of the source node. f) Addition of new links pointing from old to new nodes, on the horizontal axis we show the num. of ancestors of the target node. g) Deletion of links between old nodes, on the horizontal axis we show the num. of ancestors of the source node. h) Addition of new links between old nodes, on the horizontal axis we show the num. of ancestors of the source node. i) Addition of new links between old nodes, on the horizontal axis we show the num. of ancestors of the target node. j) Addition of links between old nodes, on the horizontal axis we show the num. of descendents of the source node. k) Deletion of links between old nodes, on the horizontal axis we show the num. of ancestors of the target node.
